# RNA modification m^6^Am: the role in cardiac biology

**DOI:** 10.1080/15592294.2023.2218771

**Published:** 2023-06-18

**Authors:** Daniel Benak, Frantisek Kolar, Lu Zhang, Yvan Devaux, Marketa Hlavackova

**Affiliations:** a Laboratory of Developmental Cardiology, Institute of Physiology of the Czech Academy of Sciences, Prague, Czech Republic; b Department of Physiology, Faculty of Science, Charles University, Prague, Czech Republic; c Bioinformatics Platform, Luxembourg Institute of Health, Strassen, Luxembourg; d Cardiovascular Research Unit, Department of Population Health, Luxembourg Institute of Health, Strassen, Luxembourg

**Keywords:** heart, epitranscriptomics, N^6^,2‘-O-dimethyladenosine, m^6^Am, N^6^-methyladenosine, m^6^A

## Abstract

Epitranscriptomic modifications have recently emerged into the spotlight of researchers due to their vast regulatory effects on gene expression and thereby cellular physiology and pathophysiology. N^6^,2‘-O-dimethyladenosine (m^6^Am) is one of the most prevalent chemical marks on RNA and is dynamically regulated by writers (PCIF1, METTL4) and erasers (FTO). The presence or absence of m^6^Am in RNA affects mRNA stability, regulates transcription, and modulates pre-mRNA splicing. Nevertheless, its functions in the heart are poorly known. This review summarizes the current knowledge and gaps about m^6^Am modification and its regulators in cardiac biology. It also points out technical challenges and lists the currently available techniques to measure m^6^Am. A better understanding of epitranscriptomic modifications is needed to improve our knowledge of the molecular regulations in the heart which may lead to novel cardioprotective strategies.

## Introduction

**Figure 1.**
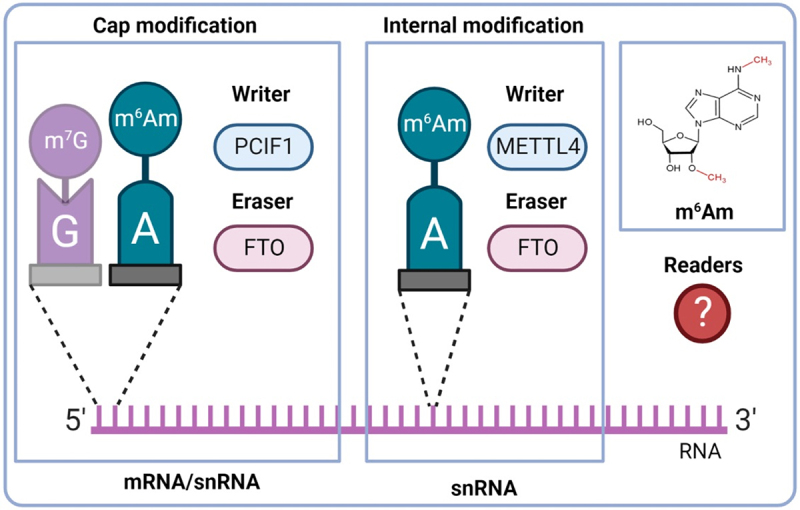
Basic overview of m^6^Am modification. A – adenosine; G – guanosine; FTO – fat mass and obesity-associated; m^6^Am – N^6^,2‘-O-dimethyladenosine; m^7^G −7-methylguanosine; METTL4 – methyltransferase-like 4; PCIF1 – phosphorylated CTD interacting factor 1.

The rapidly developing research field of epitranscriptomics has recently introduced a novel layer of gene expression regulation into cardiac biology. Over 170 chemical modifications have been found in RNA so far [1]. One of the most prevalent and characterized modifications is N^6^-methyladenosine (m^6^A) [2,3]. Similar N^6^,2’-O-dimethyladenosine (m^6^Am) is also a common form of modified adenosine (Table 1), but it is much less studied than m^6^A. This modification is formed by the methylation of a 2’-O-methyladenosine (Am). It has been described in two RNA classes: messenger RNA (mRNA) and small nuclear RNA (snRNA). In mRNA, m^6^Am is a common part of the mRNA cap and it is located at the transcription start site just next to the well-known 5-terminal modification − 7-methylguanosine (m^7^G) [4,5]. It has been found in at least 30–40% of all transcripts in vertebrate mRNA [4]. However, in specific cell lines, m^6^Am is even more dominant. For example, HEK293T cells have 92% of 5’capped mRNAs with m^6^Am and only 8% with single methylated Am [6]. The presence of m^6^Am in mRNA markedly enhances its stability due to the increased resistance of m^6^Am-modified mRNA to the mRNA-decapping enzyme DCP2 [7]. Two different isoforms of snRNAs reflecting the methylation state of A adjacent to the 5’ cap exist – m_1_ (Am) and m_2_ (m^6^Am). Cells that exhibit high m_2_ snRNA levels show modified patterns of alternative mRNA splicing [8]. m^6^Am is also present at the internal sites of snRNAs [9]. The m^6^Am/A percentage is approximately 0.01% of total RNA from human hearts [10]. This modification is dynamic, and it is regulated by proteins called writers (methylation deposition) and erasers (methylation removal) (Figure 1). No readers mediating the biological functions of m^6^Am were described so far. However, many readers binding to the more explored m^6^A are known. The question arises whether these RNA-binding proteins also recognize the similar m^6^Am modification. At present, it is known that YTH domain-containing family protein 3 (YTHDF3), one of the key m^6^A readers, does not bind m^6^Am-containing transcripts [10]. For a better understanding of m^6^Am biology, the search for m^6^Am readers is needed.

**Table 1.** Basic overview of adenosine methylations in RNA.

A	adenosine	One of the four nucleosides in RNA.
Am	2’-O-methyladenosine	Modified adenosine positioned typically next to the first nucleotide (m^7^G) in the 5’ cap. N^6^-methylation of Am forms m^6^Am.
m^6^A	N^6^-methyladenosine	A prevalent modification in mRNA, but also other types of RNA (lncRNA, rRNA, tRNA, or snRNA). The primary target of FTO in the nucleus.
m^6^Am	N^6^,2‘-O-dimethyladenosine	A modification formed by N^6^-methylation of Am in the 5’ cap (mRNA, snRNA) and also internal RNA sites (snRNA). The main target of FTO in the cytosol.
m^1^A	N^1^-methyladenosine	A modification found mainly in tRNA and rRNA. An ancillary target of FTO.

The pathological significance of m^6^Am and its regulation remains largely unknown. Recent data suggest that this modification plays a role in obesity [11], cancer [12–15], or virus–host interaction [16–18], but its function in cardiac diseases has not yet been properly addressed. However, several studies have suggested the importance of fat mass and obesity-associated protein (FTO) for cardiac function. Since this demethylase has an affinity for both m^6^A and m^6^Am, the recognition of the m^6^Am- function of FTO is important to unravel the role of m^6^Am modification in cardiac physiology and pathophysiology.

## m^6^Am writers

N^6^-methylation of Am to m^6^Am is catalysed by two known writers: phosphorylated CTD interacting factor 1 (PCIF1) and methyltransferase-like 4 (METTL4). In 2019, PCIF1 has been described as a cap-specific adenosine-N^6^-methyltransferase (also called CAPAM) which does not methylate adenosine residues in the RNA body [6,19]. However, recently it was reported that PCIF1 also has ancillary methylation activities on internal adenosines (both A and Am), although with lower affinities [20]. This writer has direct and indirect impacts on RNA stability and transcription [7,21–23]. The second m^6^Am writer – METTL4 – has been described one year later in 2020. It is responsible for internal m^6^Am formation within U2 snRNA and affects pre-mRNA splicing [24,25]. METTL4 also catalyses methylation of N^6^-methyldeoxyadenosine (6mA), a modification in mitochondrial DNA (mtDNA), particularly under stress conditions [26].

## m^6^Am erasers

So far, the only described m^6^Am eraser is FTO. Originally, FTO was described as an m^6^A demethylase [27]. However, in 2017, FTO was reported to preferentially demethylate m^6^Am rather than m^6^A [7,28]. Recently, it was suggested that the substrate preference of FTO might depend on its cellular localization, which varies between cell types. In the nucleus, FTO preferably targets m^6^A whereas cytosolic FTO demethylates especially m^6^Am [9]. The cytosolic demethylation of m^6^Am was later confirmed by others [12]. In cardiomyocytes, FTO is present in both the cytosol and the nucleus [29]. FTO demethylates m^6^Am in both mRNA and snRNA [9]. Additionally to m^6^A and m^6^Am, FTO can also target N^1^-methyladenosine (m^1^A) in transfer RNA (tRNA) [7,9].

## m^6^Am regulation in cardiac physiology and pathophysiology

**Figure 2.**
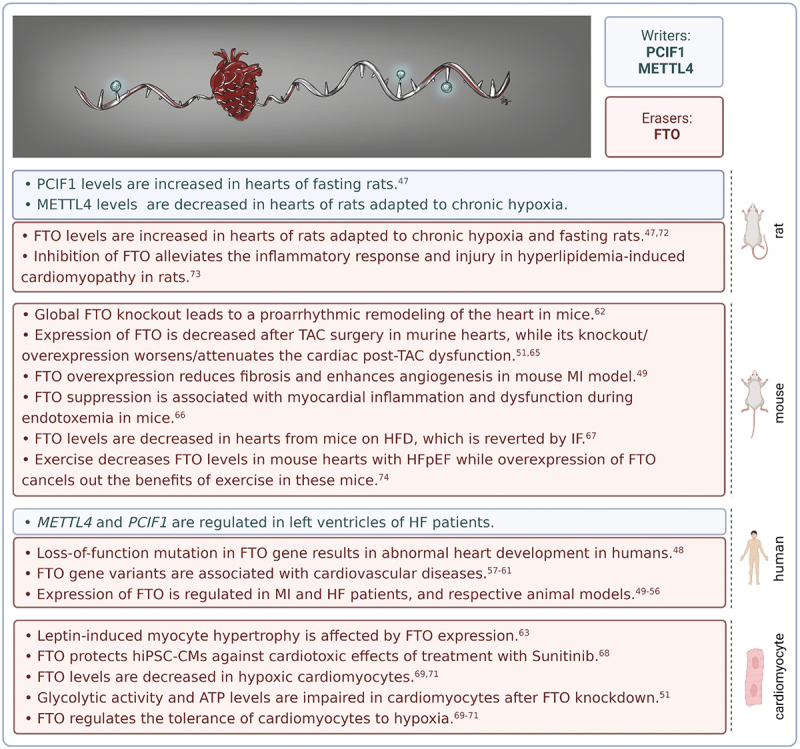
m^6^Am modification in cardiac biology. FTO – fat mass and obesity-associated; HF – heart failure; HFD – high-fat diet; hiPSC-CMs – human-induced pluripotent stem cell-derived cardiomyocytes; IF – intermittent fasting; m^6^Am – N^6^,2‘-O-dimethyladenosine; METTL4 – methyltransferase-like 4; MI – myocardial infarction; PCIF1 – phosphorylated CTD interacting factor 1; TAC – transverse aortic constriction.

Epitranscriptomics is an emerging research field in cardiovascular physiology and pathophysiology, however, attention is mainly focused on m^6^A [30–46]. The role of m^6^Am in the heart is poorly understood (Figure 2). In rRNA-depleted RNA from rat adult cardiomyocytes, the m^6^Am levels were 9-fold higher than m^6^A levels, indicating the importance of m^6^Am modification in cellular physiology (unpublished data).

## m^6^Am writers in cardiac physiology and pathophysiology

The role of m^6^Am methyltransferases in the heart is unknown. Recently, we found that METTL4 is down-regulated in the hearts of rats adapted to chronic hypoxia, a well-known cardioprotective phenomenon, while PCIF1 is not affected under these conditions (unpublished data). A regulation in protein levels of METTL4 might indicate altered mRNA splicing in hearts from chronically hypoxic animals. PCIF1, on the other hand, increased in the hearts of rats subjected to fasting, another cardioprotective intervention [47]. Publicly available RNA-seq datasets generated from human left ventricles of failing and non-failing hearts report some degree of regulation of *METTL4* and *PCIF1*. These genes were especially regulated in a large RNA-seq dataset including 356 left ventricular samples from subjects with heart failure (HF) undergoing transplantation and non-failing donors from the MAGNet consortium (Table 2). However, further research is needed to decipher the functional role of m^6^Am writers in HF, as there are no experimental data in animal models to date.

**Table 2.** Changes of m^6^Am regulators in left ventricles from heart failure patients (RNA-seq data).

Gene	Expression	Log2 Fold Change (disease/control)	P-value	FDR	Sample size	Dataset ID
*METTL4*	187	0.102	0.546	0.822	11	GSE108157
*PCIF1*	917	0.046	0.662	0.879	11	GSE108157
*FTO*	2132	−0.135	0.193	0.578	11	GSE108157
*METTL4*	256	−0.123	0.142	0.223	64	GSE116250
*PCIF1*	**988**	**0.336**	**7.48E–08**	**8.65E–07**	**64**	**GSE116250**
*FTO*	1703	0.077	0.151	0.235	64	GSE116250
*METTL4*	254	−0.151	0.105	0.213	30	GSE135055
*PCIF1*	845	0.025	0.708	0.807	30	GSE135055
*FTO*	1327	−0.039	0.640	0.755	30	GSE135055
*METTL4*	**319**	**−0.138**	**3.95E–03**	**0.007**	**356**	**GSE141910**
*PCIF1*	**725**	**0.206**	**6.27E–07**	**1.88E–06**	**356**	**GSE141910**
*FTO*	1156	−0.054	0.238	0.293	356	GSE141910
*METTL4*	81	0.051	0.709	0.847	23	GSE150736
*PCIF1*	268	0.193	0.0512	0.190	23	GSE150736
*FTO*	690	0.133	0.169	0.387	23	GSE150736
*METTL4*	174	0.061	0.552	0.750	15	GSE203160
*PCIF1*	801	−0.152	0.037	0.145	15	GSE203160
*FTO*	2119	0.004	0.942	0.974	15	GSE203160

Comparison of the expression of m^6^Am regulators in the left ventricles of heart failure patients and healthy controls based on analysis of 6 RNA-seq experiments obtained from the GEO database. Changes significant according to FDR (false discovery rate) value are in bold. FTO – fat mass and obesity-associated; METTL4 - methyltransferase-like 4; PCIF1 - phosphorylated CTD interacting factor 1.

## m^6^Am erasers in cardiac physiology and pathophysiology

Undoubtedly, the best-studied m^6^Am regulator is FTO. This eraser is essential for the normal development of the cardiovascular system in humans. Loss-of-function mutation in the human *FTO* gene caused several heart defects (ventricular septal defect, atrioventricular defect, patent ductus arteriosus) and hypertrophic cardiomyopathy [48]. Regulated expression of FTO was observed in myocardial infarction (MI) and HF patients and respective animal models [49–56]. Interestingly, RNA-seq datasets that revealed regulation of m^6^Am writers in HF patients did not show significant changes in *FTO* expression (Table 2). Since HF is a heterogeneous clinical syndrome with complex pathophysiology, more studies with comparable methodology are necessary to reliably assess the role of FTO in different HF aetiologies. Gene variants of *FTO* were linked with various cardiovascular diseases, including MI, acute coronary syndrome, and an increased risk of rejection in heart transplant patients [57–61].

Global knockout (KO) of the mouse *Fto* gene led to a proarrhythmic remodelling of the heart. KO mice displayed higher heart rate and heart rate variability compared to their wild-type counterparts. Moreover, they were more vulnerable to stress-induced tachyarrhythmias, their ventricular repolarization was altered, and they also developed myocardial hypertrophy [62]. Studies on cultured neonatal rat cardiomyocytes showed that myocyte hypertrophy can be caused by a leptin-induced increase in FTO expression and that FTO knockdown with siRNA abrogated this event [63]. Mice lacking FTO specifically in cardiomyocytes showed worsened cardiac phenotype characterized by reduced ejection fraction and increased dilatation upon transverse aortic constriction (TAC) surgery, an experimental model for pressure overload-induced cardiac hypertrophy and HF [64]. TAC surgery itself led to a decreased expression of FTO while FTO overexpression attenuated the cardiac post-TAC dysfunction [51,65]. FTO suppression was also associated with myocardial inflammation and dysfunction during endotoxemia in mice [66]. In murine isolated primary cardiomyocytes, FTO knockdown impaired the glycolytic activity and decreased ATP levels [51].

Recently, it has been shown that FTO plays a role in cardioprotection. Heart tissues from mice on a high-fat diet (HFD) contained decreased levels (both mRNA and protein) of FTO, which were reversed by intermittent fasting, a nutritional approach improving HFD-induced obesity cardiomyopathy [67]. These changes in FTO expression were also associated with corresponding changes in m^6^A/m levels (detection of m^6^A which does not differentiate between m^6^A and m^6^Am). Ma et al. [68] showed that FTO protected human-induced pluripotent stem cell-derived cardiomyocytes (hiPSC-CMs) against treatment with Sunitinib, a tyrosine kinase inhibitor with cardiotoxic effects. Importantly, FTO also affected the tolerance of cardiomyocytes to hypoxia, a key parameter in the possible prevention of ischaemic heart disease. FTO was poorly expressed in human cardiomyocyte cell line AC16 exposed to acute hypoxia/reoxygenation (H/R) and its up-regulation improved cell viability after H/R insult [69]. Likewise, in mouse cardiomyocytes, FTO overexpression inhibited apoptosis induced by acute H/R while FTO knockdown had the opposite effect [70]. Moreover, Ke et al. [71] observed the downexpression of FTO in mouse hearts and isolated cardiomyocytes subjected to ischaemia-reperfusion and acute H/R insults, respectively. FTO overexpression then led to attenuation of the H/R-induced apoptosis and inflammation in these cells. In our lab, we found that administration of FTO inhibitor MO-I-500 increased the lactate dehydrogenase release (which indicates cell damage) and decreased cell viability in rat cardiomyocytes under acute hypoxic conditions (unpublished data). Adaptation of rats to 3 weeks of chronic hypoxia and 3-day fasting, both cardioprotective regimes, was associated with increased myocardial levels of FTO, which might thus participate in the induction of the protection [47,72]. FTO overexpression also reduced fibrosis and enhanced angiogenesis in mouse models of myocardial infarction [49].

Contrary to the data showing the beneficial effects of FTO on the heart, Yu et al. [73] reported that inhibition of FTO using LuHui Derivative (LHD) compound alleviated the inflammatory response and injury in hyperlipidaemia-induced cardiomyopathy in rats. Similarly, mice with heart failure (HFpEF) exhibited decreased FTO levels in the heart after physical training while overexpression of FTO abolished the health benefits of exercise in these mice by promoting myocyte apoptosis, myocardial fibrosis, and myocyte hypertrophy [74].

It is worth mentioning that FTO regulations in the heart might be age-dependent. According to Su et al. [75], FTO levels dropped in elderly murine hearts in response to acute myocardial ischaemia/reperfusion injury while in young hearts it remained unaffected. FTO levels also changed during postnatal development [76]. Moreover, the hearts of newborn male rats exhibited higher FTO protein levels than females, suggesting possible sex-dependent differences in m^6^Am regulations [76].

These data show that FTO can have both beneficial and detrimental effects on the heart. However, current FTO studies have focused on m^6^A and have not included m^6^Am. Since many m^6^A detection methods do not distinguish between these two modifications, the potential involvement of m^6^Am could be masked. Separating the m^6^A-specific and m^6^Am-specific mechanisms of action in the future is essential for understanding the role of m^6^Am in cardiac biology. In rRNA-depleted RNA isolated from rat cardiomyocytes, the m^6^Am levels are 9-fold higher compared to m^6^A. Nevertheless, according to RM2Target (database for targets of writers, erasers, and readers), data on the m^6^Am-targets of FTO in the heart are virtually missing [77].

## m^6^Am regulators as pharmacological targets

At present, several inhibitors of demethylase FTO are available. In 2012, the natural product rhein was identified as the first cell-active inhibitor of FTO that was capable to increase the modification level of m^6^A in mRNA [78]. Two years later, compound MO-I-500 was introduced as another specific inhibitor of this demethylase [79]. A known anti-inflammatory drug meclofenamic acid (MA) was also identified as a highly selective inhibitor of FTO [80]. Treatment of HeLa cells with either MO-I-500 or MA resulted in the elevation of m^6^A levels in mRNA [79,80]. Fluorescein and its derivatives were introduced as bifunctional molecules that can be used for FTO inhibition or labelling [81]. Selective inhibition of FTO was also achieved by other small molecule inhibitors, such as FB23, FB232, CS1, CS2, Dac51, LHD, FTO-02, or FTO-04 [73,82–89]. Since FTO is a non-specific demethylase, it is expectable that its inhibition may affect levels of all its substrates. For instance, treatment of glioblastoma stem cells with FTO-04 resulted in increases in both m^6^A and m^6^Am levels [87]. Unfortunately, current FTO inhibitors are not suitable for clinical use due to either low target selectivity or pharmacokinetic properties [87]. Compounds affecting other m^6^Am regulators were not described so far. Future identification of effective pharmacological agents targeting m^6^Am regulation is important for the development of novel therapies for cardiac diseases.

## m^6^Am research methods

**Figure 3.**
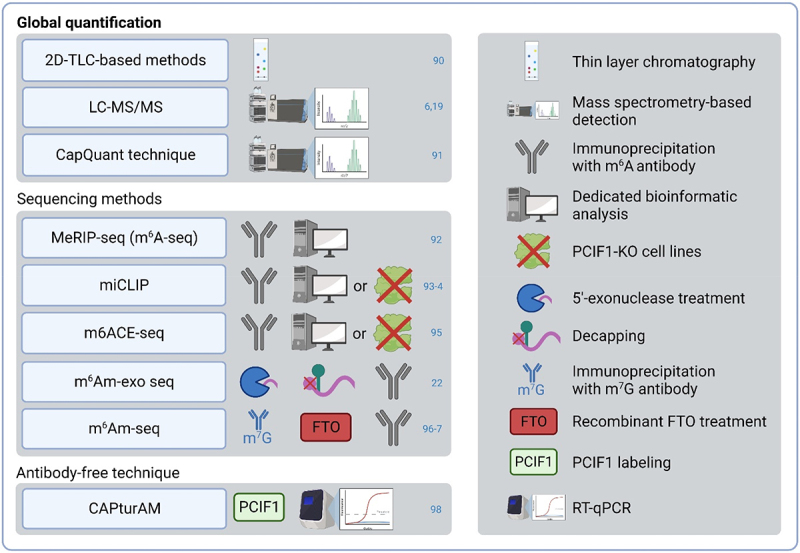
m^6^Am detection methods. 2D-TLC – two‐dimensional thin‐layer chromatography; FTO – fat mass and obesity-associated protein; LC-MS/MS – liquid chromatography-tandem mass spectrometry; m6ACE-seq – m^6^A-crosslinking-exonuclease-sequencing; m^6^Am-exo-seq – m^6^Am-exonuclease-assisted-sequencing; m^6^Am-seq – m^6^Am-sequencing; MeRIP-seq – methyl RNA immunoprecipitation and sequencing; miCLIP – m^6^A individual-nucleotideresolution crosslink and immunoprecipitation; PCIF1 – phosphorylated CTD interacting factor 1; RT-Qpcr – reverse transcription quantitative real-time polymerase chain reaction.

To determine the effects of m^6^Am, it is necessary to properly distinguish m^6^Am from similar m^6^A. Antibodies used in the m^6^A detection bind both m^6^A and m^6^Am and therefore incorrectly annotate also m^6^Am as m^6^A [7]. Another problem is that m^6^Am regulators are not m^6^Am-specific. METTL4 can also catalyse methylation of 6mA and FTO can demethylate m^6^A and m^1^A, so both of these regulators might affect the cardiac biology also in an m^6^Am-independent manner. Thus, future studies should focus on the differentiation of m^6^A and m^6^Am regulations. Various novel techniques can deal with the similarity of these two modifications and distinguish m^6^Am from m^6^A (Figure 3).

Similarly to other modifications, relative quantification of m^6^Am levels can be achieved by a thin layer chromatography (TLC) method or mass spectrometry-based detections such as liquid chromatography with tandem mass spectrometry (LC-MS/MS) or CapQuant technique [6,19,90,91]. Moreover, several high-throughput sequencing methods are used for m^6^Am profiling. The m^6^A antibodies used in these techniques do not differentiate between m^6^A and m^6^Am. Various approaches have been used to overcome this issue. For example, MeRIPseq (also called m^6^A-seq), miCLIP, or m6ACE-seq require either a dedicated bioinformatics analysis (as m^6^Am is present at the 5’end of mRNAs while m^6^A is enriched in its internal regions) or signal depletion of m^6^Am in the PCIF1-KO cell lines to reveal m^6^Am profiles [92–95]. m^6^Am-exo-seq relies on the elimination of uncapped RNA fragments (with m^6^A modification), resulting in the enrichment of 5’-end RNA fragments containing m^6^Am. Subsequent decapping of 5’-end RNA fragments facilitates m^6^Am recognition by m^6^A antibody [22]. Likewise, m^6^Am-seq utilizes cap-m^7^G immunoprecipitation to purify 5’-end RNA fragments and then uses recombinant FTO treatment to erase m^6^Am before sequencing. Since FTO has a higher affinity to m^6^Am compared to m^6^A *in vitro*, the comparison of FTO-treated and untreated samples allows the specific identification of m^6^Am sites [96,97]. Recently, CAPturAM, an antibody-independent method to selectively enrich and detect physiological targets of PCIF1 has been introduced. This technique is based on RNA labelling with recombinantly produced PCIF1 and assessment by RT-qPCR. CAPturAm allows the identification of the transcription start nucleotide N^6^-methylation status by comparing enrichment between WT and PCIF1-KO cells [98]. In addition to these experimental methods, several computational approaches were introduced to accurately identify m^6^Am sites based on sequence-derived data, such as m6AmPred, MultiRM, or DLm6Am [99–101].

## Conclusion

m^6^Am is one of the most prevalent modifications of RNA. Its regulation might have a profound effect on cardiac physiology, yet the knowledge of its functional role in cardiac disease development as well as its potential value as a therapeutic target and biomarker deserves further investigation. The epitranscriptomics field remains uncharted territory that might reveal clinically relevant discoveries in the future.

## Data Availability

The RNA-seq data analysed in this study (Table 2) are available at the Gene Expression Omnibus (GEO) data repository, which is accessible at https://www.ncbi.nlm.nih.gov/geo/.
